# Feature Extraction for Track Section Status Classification Based on UGW Signals

**DOI:** 10.3390/s18041225

**Published:** 2018-04-17

**Authors:** Lei Yuan, Yuan Yang, Álvaro Hernández, Lin Shi

**Affiliations:** 1 Electronics Department, Xi’an University of Technology, Xi’an 710048, China; yuanleixut@126.com (L.Y.); lincoding@163.com (L.S.); 2 Electronics Department, University of Alcala, Alcalá de Henares, Madrid 28805, Spain; alvaro.hernandez@uah.es

**Keywords:** track status classification, ultrasonic guided wave (UGW), feature extraction, temporal and spatial dependencies, deep learning algorithm

## Abstract

Track status classification is essential for the stability and safety of railway operations nowadays, when railway networks are becoming more and more complex and broad. In this situation, monitoring systems are already a key element in applications dedicated to evaluating the status of a certain track section, often determining whether it is free or occupied by a train. Different technologies have already been involved in the design of monitoring systems, including ultrasonic guided waves (UGW). This work proposes the use of the UGW signals captured by a track monitoring system to extract the features that are relevant for determining the corresponding track section status. For that purpose, three features of UGW signals have been considered: the root mean square value, the energy, and the main frequency components. Experimental results successfully validated how these features can be used to classify the track section status into free, occupied and broken. Furthermore, spatial and temporal dependencies among these features were analysed in order to show how they can improve the final classification performance. Finally, a preliminary high-level classification system based on deep learning networks has been envisaged for future works.

## 1. Introduction

Railway transportation has improved rapidly in recent years, achieving significant and widespread use in China, especially with the development of high-speed railway lines. As one of the major components of railway transportation systems, tracks have been the subject of increased engineering effort, not only in terms of design, manufacturing and deployment, but also with regard to monitoring and safety issues. As a consequence of the long-term strain on tracks due to the existing external forces [1,2], track degradation and breakage have become very important concerns. Breakage poses a great threat to railway transportation security; in the best cases it can cause train delays, but at worse it can lead to accidents and casualties. For this reason, the proposal and design of accurate and real-time track status monitoring systems is still a relevant topic [3].

Different methods have already been applied to monitor track status. Among these, track circuits are the most typically used and extended method. In their basic configuration, a track circuit provides a certain energy to each rail, so a relay coil wired across rails can report the track condition based on its own status (energized or de-energized). However, track circuits are significantly affected by the ballast parameters, and this may lead to the appearance of false alarms with regard to short-circuits [4,5]. In [6], the Salient System Company proposed a method, which laid out sensors every 30~60 m along the track, so every sensor can detect the track strain and temperature, as well as report data to a monitoring center through a wireless module. This method is suitable for new railway lines to be built in the coming years, but not for existing ones, since it is complicated to determine the zero-strain point (required by the algorithm) for a track already installed and in operation. In [7], a detection method based on using the unbalanced traction return currents is proposed, although it is important to note that this approach requires a train passing through the section under analysis to detect any breakage status. 

As a medium of solid sound propagation, tracks often have suitable acoustic features. When ultrasonic waves are confined within the boundaries of a bar or tubular medium, the boundary produces repeated reflections on the ultrasonic waves, thus forming ultrasonic guided waves (UGW), which consist of a longitudinal wave, a surface wave, a lamb wave, as well as other basic ultrasonic types in various ways. Compared to the traditional single-wave energy centralized ultrasonic waves, UGW provide a relatively low detection frequency, and a long transmission and detection distance [8,9].

Taking that into account, in 2002, the RailSonic Company in South Africa took advantage of these characteristics and proposed a novel detection system based on UGW. The system mainly consists of ultrasonic transducers, transmitters and receivers, as well as the corresponding communication modules, and tracks play the role of the transmission channel in the proposal [10]. This method uses a mechanical wave as the detection signal, thereby it is not affected by any traction current or track electrical parameter. Furthermore, the installation and maintenance of this type of device has been proven to be convenient [1] and additionally, the power consumption is also relatively low. The aforementioned advantages provided by this detection method have led to the fact that many researchers and studies have been focused recently on detection systems based on UGW signals [11,12].

In [13], different experiments were carried out to test the suitable driving frequency and transmission distance for the UGW signal and a novel breakage detection method using the echo impulse was also proposed [14]. UGW signals were also studied in [15], where a detection method based on a fixed threshold was developed. Furthermore, a monitoring system for continuous welded railways (CWR) based on UGW signals was proposed in [16,17], where the electronic architecture involved in the experimental implementation included an ARM processor and a field-programmable gate array (FPGA) device, thus achieving two km long track sections. Similarly, time-frequency analysis methods, such as the Short-Time Fourier Transform (STFT) and Pseudo Wigner Ville Distribution (PWVD) have focused on the attenuation characteristics of UGW signals as well [18,19]. On the other hand, in [20] a signal processing approach with a smooth empirical mode decomposition applied to UGW signals was proposed. In [21,22], UGW signals were applied to pipeline flaw identification. An amplitude dispersion compensation for damage detection using UGW signals was studied in [23,24], and a study of non-detection zones in conventional long-distance UGW inspection on square steel bars was presented. Finally, various previous works have also dealt with mode identification and denoising methods for UGW signals [25,26], as well as with modelling of a UGW transducer [27]. However, ways to extract the features and regular rules of UGW signals in the track status monitoring system are still very important for track status detection. 

This work proposes a method to extract features from the UGW signals involved in a monitoring system, so they can be applied to distinguish three different track statuses; free, occupied and broken. The main contribution is the extraction of these three features from the UGW signals; the RMS value and the energy in the time domain, as well as the frequency component with the highest amplitude in the frequency domain. Since these features often follow a certain variation or trend over time, the temporal dependencies have been also considered. Furthermore, taking into account that a railway line consists of a set of successive track sections, where the status of one is also related to the status of previous and later ones, the existing spatial dependencies between successive track sections have been studied. The analysis of the aforementioned features, together with the temporal and spatial dependencies along the successive track sections of a railway line, allow the track section status classification to be carried out correctly. It is worth noting that the use of UGW-based detection systems in this type of application is still an open and challenging question, where certain drawbacks should be addressed in the coming years. The propagation of UGW signals can be affected by environmental factors, and this can lead to different issues in the practical implementation, with high false alarm rates in the track status classification. Therefore, in long-term, complex and large-scale railway transportation networks, it is necessary to apply certain high-level techniques, such as deep learning algorithms [28], which can deal with varying situations and conditions in a reliable way. Some experimental tests have verified the correctness and suitability of the selected features. The rest of the manuscript is organized as follows: the existing UGW-based track detection system is described in Section 2; Section 3 explains the proposed feature extraction scheme; Section 4 shows some experimental results regarding the railway status classification; future works are proposed in Section 5, and finally, conclusions are discussed in Section 6. 

## 2. UGW-Based Track Detection System

**Figure 1:**
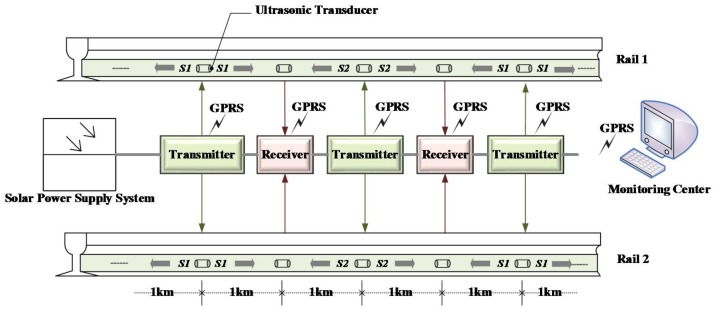
Typical structure for the UGW-based track monitoring system.

An already available UGW-based track monitoring system has been used here to estimate the track status, and, especially to detect possible breakages [29,30]. The general overview of the system is depicted in Figure 1; it includes a monitoring center and a set of nodes distributed along the railway. Every node mainly involves a solar power supply system, a transmitter or a receiver (depending on the type of node), as well as a wireless communication module. The solar power supply system provides the power required by each node, and transmitters and receivers are placed alternately every 1 km. Each transmitter or receiver is connected to the rail waist through two UGW transducers (one per rail), again with a distance of 1 km from each other. The rail segment between a transmitter and the following receiver is known as a track section. Through a wireless communication module, based on General Packet Radio Service (GPRS), information gathered for every track section can be sent to a remote monitoring center. 

**Figure 2:**
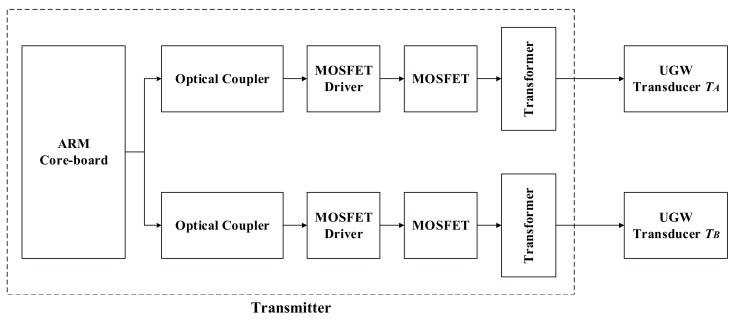
Block diagram for the UGW transmitter.

**Figure 3:**
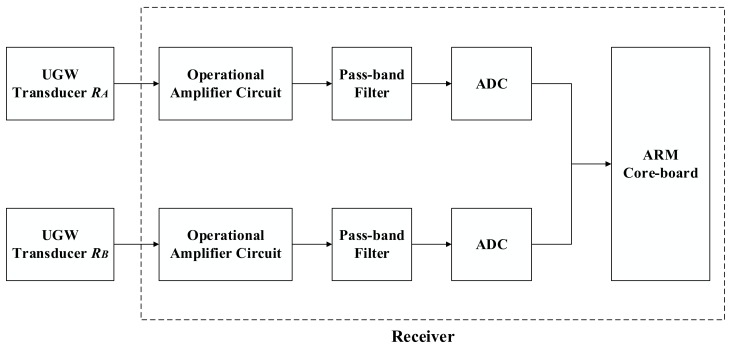
Block diagram for the UGW receiver.

The block diagrams for both the transmitter and the receiver can be observed in Figure 2 and Figure 3, respectively. The transmitter structures are denoted within the dotted box, where the available ARM core is in charge of generating the driving square signals. Then, these signals are sent to two identical processing lines. By means of an optical coupler, the aforementioned square signals are connected to a MOSFET driver and a transformer. The final output signals are used to energize the UGW transducers and generate the corresponding UGW signals. The transducer *T_A_* is connected to rail no. 1, whereas the *T_B_* transducer is connected to rail no. 2. At a distance of 1 km from there, the receiver is placed, according to the scheme shown in Figure 3. The corresponding UGW signals are captured by transducers *R_A_* and *R_B_*, which are connected to rail no. 1 and rail no. 2, respectively. Both received signals are processed and acquired in two different processing lines. These lines consist of an amplification stage (based on an operational amplifier) and a band-pass filter to discard undesired noise. Through the Analog-Digital Converter (ADC), the digital signals are sent to the ARM core for further processing. By checking the time and frequency characteristics of the received signals over a certain interval containing the UGW signals transmitted by *T_A_* and *T_B_*, the status of the corresponding track section under analysis can be determined.

**Figure 4:**
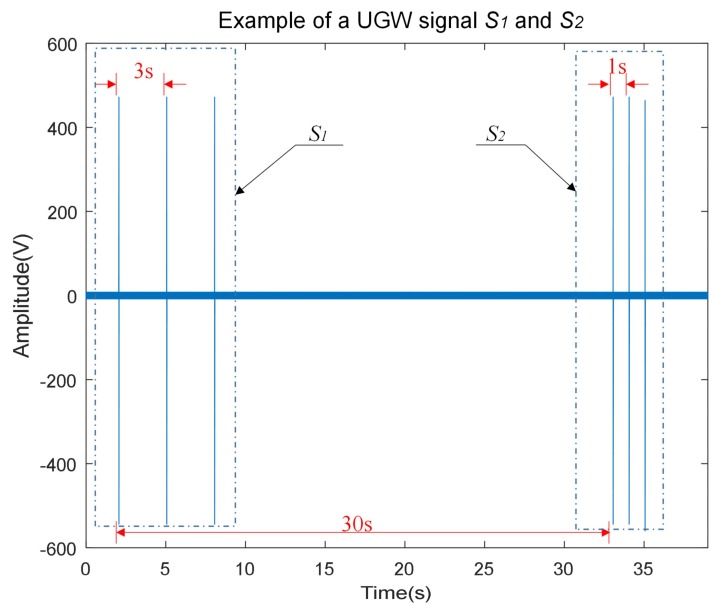
Example of UGW signals *S*_1_ and *S*_2_.

Under the ordinary working conditions of the system, all the odd-numbered transmitters simultaneously drive their ultrasonic transducers *T_A_* and *T_B_* to transmit their own characteristic signal *S*_1_ to both sides, left and right, along the rails. After thirty seconds, all the even-numbered transmitters proceed in the same way to generate their own signal *S*_2_ to both sides. Therefore, transmitters *T_A_* and *T_B_* can alternately emit the corresponding signals *S*_1_ and *S*_2_ every 30 s. Examples of signals *S*_1_ and *S*_2_ captured in the transmitter, after the transducers are plotted in Figure 4, where common amplitudes are around 500 V. Both signals *S*_1_ and *S*_2_ consist of three typical trains of UGW pulses (every train formed by 30 square pulses with a length of 33.33 μs). Though the waveform of both signals is similar, the interval among these three trains of UGW pulses is 3 s for the signal *S*_1_, whereas it is only 1 s in the case of *S*_2_. Since the receivers and transmitters are fully synchronized, receivers can detect which signal, *S*_1_ or *S*_2_, is received at any moment and then manage the ADC to acquire the corresponding signals every 3 s or 1 s, respectively, all of them inside a time window of 20 ms to include a train of UGW pulses. Note that the sampling frequency in the ADC is *f_s_* = 250 kHz. 

According to the experiments carried out on the BART railway line [13], as well as those on the Spoornet track section [10], the transmission rail channel affects the UGW signals in different ways, depending on the main frequency components used for that propagation. For the same propagation distance, tracks often have a relative low impedance in the range of the 20 kHz–40 kHz UGW frequency band, thus implying that UGW signals get less attenuation and achieve longer distances, so the received signals can be detected more easily. Due to this, the main frequency component considered for the UGW signals *S*_1_ and *S*_2_ was 30 kHz. This also defines the central frequency for the band-pass filter in Figure 3 at 30 kHz, with a bandwidth of 6 kHz. 

When a track section is free, the corresponding receiver can acquire the aforementioned signals *S*_1_ and *S*_2_. On the contrary, the receiver has difficulty detecting the arrival of these identifying signals when a train is in a certain track section, or if a track breakage occurs. Nonetheless, the receiver can acquire the signals again once the train leaves the corresponding track section, whereas in case of breakage, the characteristic signals will still be missing over time, no matter the presence of a train in the track section. In any case, receivers report information to a remote monitoring center through a wireless communication module, warning of any possible breakage or occupancy alarm, so, if necessary the follow-up maintenance can be scheduled and carried out, avoiding any further impact on train circulation.

## 3. Proposed Track Status Feature Extraction

With regard to any high-level techniques, such as deep learning networks, which can be applied to the classification of the track status based on UGW signals, they require the availability of certain information about how identifying features extracted from UGW signals behave under the considered conditions for a track section: free, occupied and broken. In that way, the more relevant the feature differences between different track status are, the higher the success rate for the status classification becomes. Considering all these aspects, a track status feature extraction based on UGW signals is proposed hereinafter, keeping in mind the improvement of the classification accuracy.

As has already been mentioned, in the proposed UGW-based track monitoring system, a receiver acquires the transmitted signals *S*_1_ and *S*_2_ coming from the next emitters in both directions. The corresponding track status can be estimated by analyzing those signals. For every section under analysis, the track status can be mainly classified into three types: free, occupied and broken. For that purpose, the received UGW signals are processed over a time window of 20 ms. The status of the corresponding track section is proposed to be determined by extracting the following three identifying features: the root-mean-square (RMS) value *V_RMS_* and the energy *E* from the received signals, and their more relevant frequency component *f_P_* (corresponding to the amplitude peak). For clarity’s sake, it is worth noting that both rails, no. 1 and no. 2 have the same setup, so only those signals coming from rail no. 2 will be described in detail here for the three considered cases (free, occupied and broken), thus assuming that similar conclusions can be extended to the other rail. 

It is important to note that the following description of the proposed feature extraction will be illustrated by means of experimental signals acquired by the corresponding electronic equipment installed on the railway line from Baoji to Chengdu (China). The distance between transmitters and receivers is 1 km, and the weather conditions were relatively dry. Also, the UGW signals adopted in this work were designed to reach as far as 1.5 km, thus ensuring that receivers can only capture UGW signals coming from their two immediate transmitters.

### 3.1. Track Status: Free

**Figure 5:**
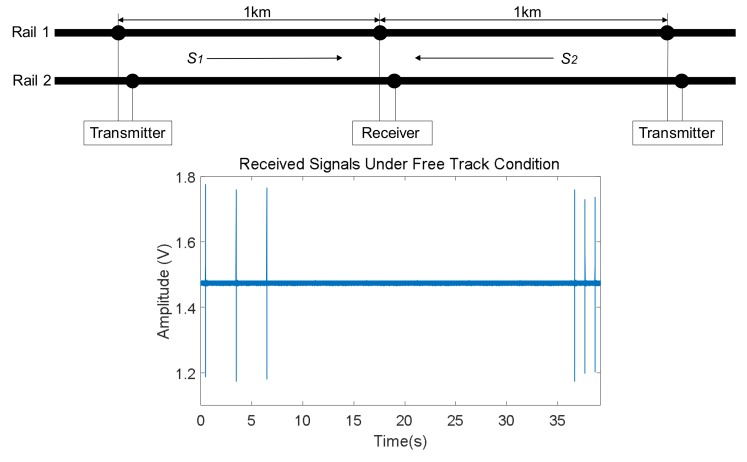
Example of a reception in rail no. 2 including both signals *S*_1_ and *S*_2_ for a free track section.

**Figure 6:**
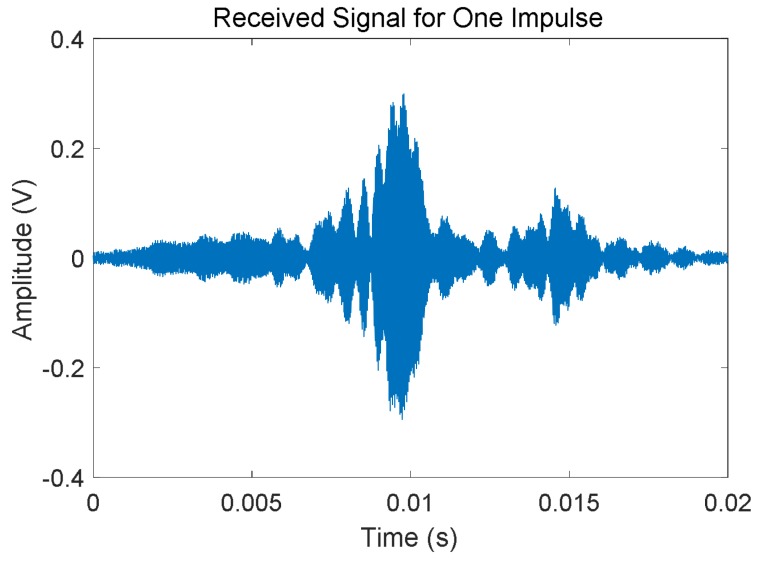
Zoom of an UGW train of pulses received for a free track section.

For many railway lines, the free track status is often the most common one, which means tracks are intact and there are no trains occupying the corresponding sections. Then, under this track status trains are allowed to go into those sections without any potential security risk. Figure 5 shows an example case for one period of the received signals *S*_1_ and *S*_2_ acquired by the ADC for rail no. 2, coming from both the left and right neighboring sections. As can be observed, whether the track is free or not, the receiver in the middle can acquire the characteristic UGW signal *S*_1_ from the left side, as well as the signal *S*_2_ from the right one. Note that these two signals are still 30 s apart. Furthermore, the 20 ms long window used to deal with a train of UGW pulses is also plotted in Figure 6 (after removing the DC offset). This signal is processed in order to determine the aforementioned identifying features (*V_RMS_*, *E*, *f_P_*) for the estimation of the current status in the involved track section.

In this work, the RMS feature *V_RMS_* of the received signal is used to quantify the voltage variations, and its value is calculated over a one-cycle-long window (20 ms). Note that, at the receiver end, the UGW signals processed by the ARM core have been sampled at *f_s_* = 250 kHz, so a window of UGW pulses with a length of 20 ms is composed of *N* = 5000 discrete samples. In this case, the RMS value *V_RMS_* for a train of UGW pulses can be defined by (1). 

(1) $$V_{RMS}=\sqrt{\frac{1}{N}\sum_{n=1}^{N}v_{n}^{2}}$$

On the other hand, the time-domain energy *E* for a train of UGW pulses is also calculated according to (2), for the 20 ms long UGW signal

(2) $$E=\sum_{n=1}^{N}{\left|v_{n}\right|}^{2}$$

**Figure 7:**
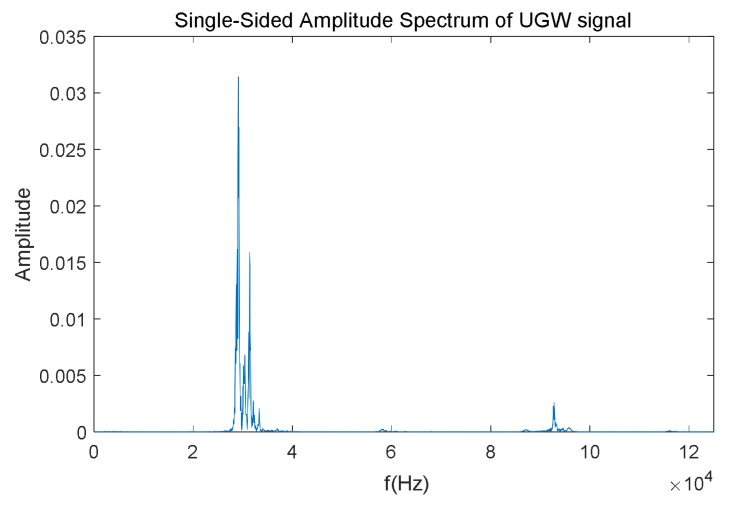
Example of the FFT for the train of UGW pulses shown in Figure 6.

Finally, the frequency analysis is carried out by computing an FFT (Fast Fourier Transform) algorithm. As an example, the FFT outcome for the UGW signal with a length of 20 ms shown in Figure 6 is represented in Figure 7. As expected, the dominant frequencies are mainly focused around 30 kHz; furthermore, though there is a peak at 92 kHz, its amplitude is low, and this is caused by the mix of traction currents and environmental noise In this work the focus was on the highest frequency component *f_P_* and its amplitude *P* in the received signal, about 30 kHz for the example shown in Figure 7.

### 3.2. Track Status: Occupied

**Figure 8:**
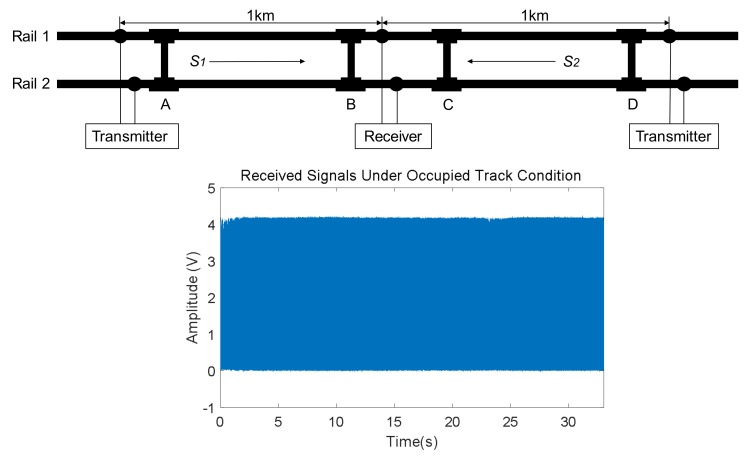
Example of received UGW signals in rail no. 2 for an occupied track section.

The status of a track section is occupied when there is a train rolling through it, so no other trains are allowed to use it. As the train gets closer to the track section under analysis, the typically received UGW signals are gradually masked by the mechanical noise produced by the train wheels and axles. In the same way, as soon as the train leaves the mentioned track section, the UGW signals start to gradually reappear. When the track is occupied by a train, the UGW signals are completely lost in the added noise, as can be observed in the corresponding plot in Figure 8. 

**Figure 9:**
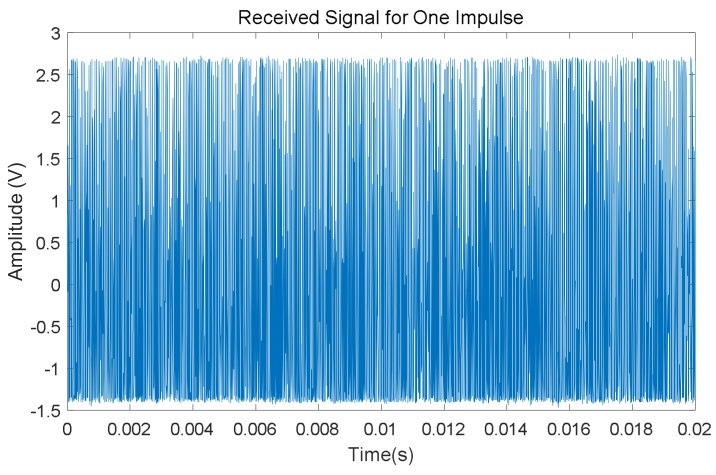
Example of a train of UGW pulses masked under noise for an occupied track section.

**Figure 10:**
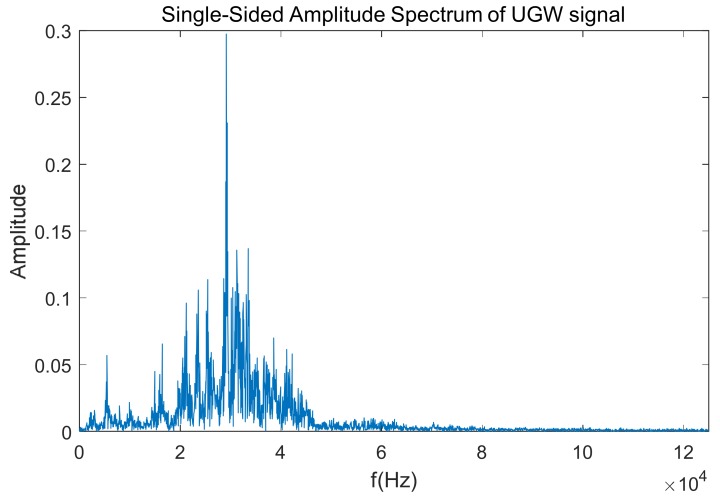
Frequency-domain representation of a train of UGW pulses for an occupied track section.

In the case of an occupied track section, though all the UGW signals are masked by noise, the receiver can still obtain the 20 ms long signals, including the transmitted train of pulses, thanks to the existing synchronization between transmitters and receivers. For example, Figure 9 shows an interval of 20 ms, which contains the masked UGW signals for an occupied track section, whereas the same signals in the frequency domain have been plotted in Figure 10. Due to the 30 kHz band-pass filter, the frequency spectrum is still focused around that value; nevertheless, when compared to the frequency spectrum in Figure 7, the energy is more spread in the frequency domain under the occupied situation.

For an occupied track section, the RMS value *V_RMS_* and the signal energy *E* in the time domain are also calculated, although both values may be useless due to the influence of the noise produced by the train on the values. Besides, both values are much higher than those for a free track section. On the other hand, because of the impact from the band-pass filter, the frequency *f_p_*, at which the maximum *P* is achieved in the spectrum of the received signal, is similar to that estimated for the free section.

### 3.3. Track Status: Broken

**Figure 11:**
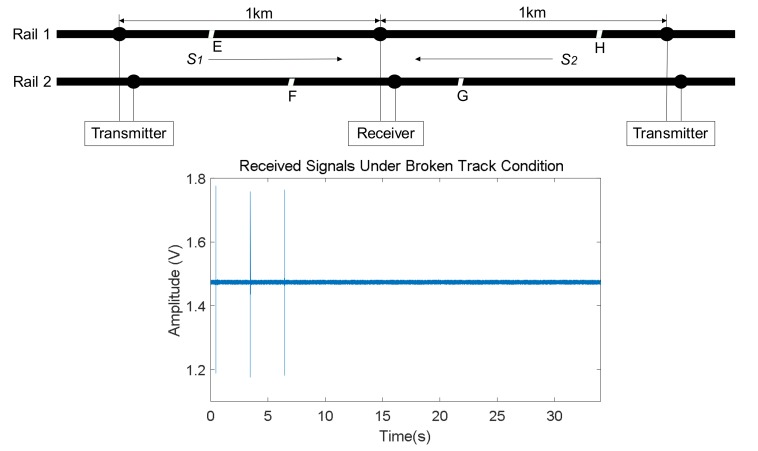
Diagram and example of UGW signals received in rail no. 2 for a broken track section.

**Figure 12:**
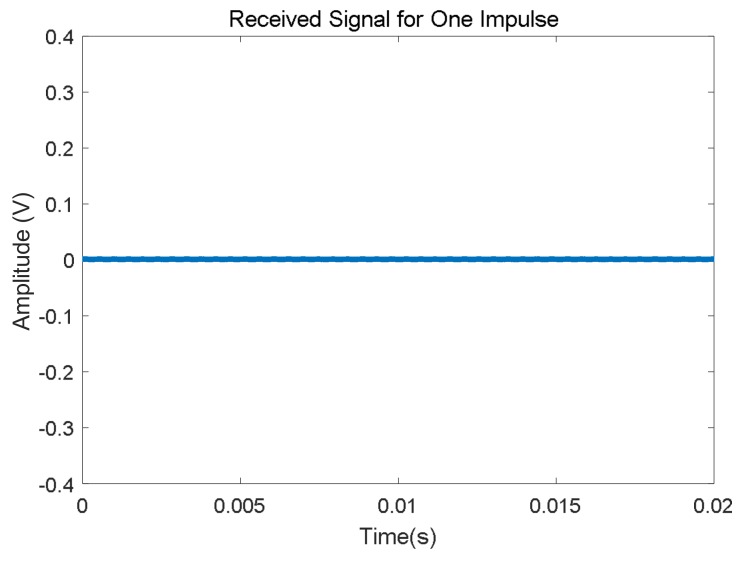
Zoom for a 20 ms window in the received UGW signal where pulse S_2_ should be included, considering a breakage G.

**Figure 13:**
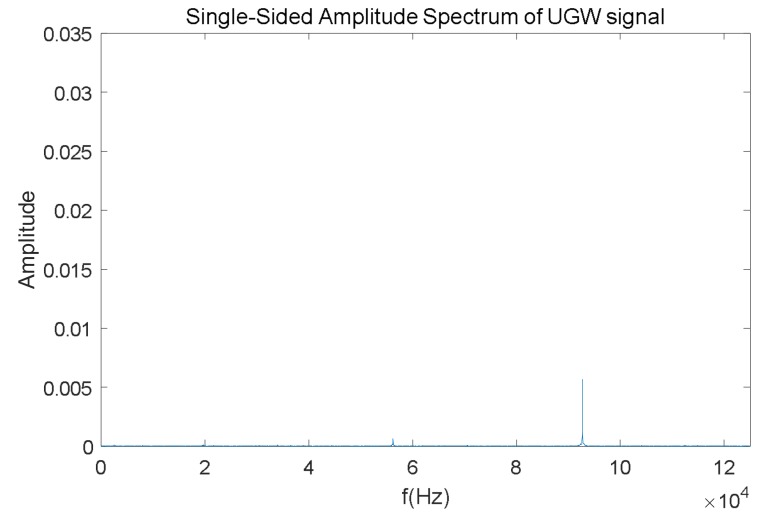
Frequency-domain representation for a train of UGW pulses S_2_ in a broken track section, according to Figure 11.

The UGW signals are attenuated at a certain level when propagating through rails. Furthermore, in the case of a breakage happening in any rail, the energy of the UGW signals is partially lost and reflected, thus causing a significant energy attenuation in the transmissions and the maximum propagation distance is consequently shortened. This implies that the receivers will not be able to properly detect the transmitted UGW signals. Figure 11 depicts an example where four possible breakages, E, F, G and H have been considered. Due to these, the UGW signals in the corresponding section are lost. In the following graph, the UGW signals captured for rail no. 2 are represented, and only breakage G has been taken into account. Thereby, the three trains of UGW pulses *S*_1_ coming from the left transmitter are visible, whereas the pulses S_2_ from the right transmitter are practically negligible (see Figure 12). In the same way as before, if the FFT is applied to a 20 ms window of the received signals, where pulses *S*_2_ should appear but will not, it is possible to check in Figure 13 that no frequency components are available in the same range around 30 kHz, with only one maximum at 92 kHz. 

Finally, for a broken track section, both values *V_RMS_* and energy *E* in the time domain are much lower than those for a free track section. Furthermore, the corresponding frequency *f_p_* for the largest component *P* in the signal spectrum is roughly at 92 kHz, similar to the noise component in a free track section, so there is no trace about the transmitted signals *S*_1_ and *S*_2_ in the frequency domain. 

Taking into account the comments above, it is possible to observe that the RMS value *V_RMS_*, as well as the energy feature *E*, have the same variation trend for the three considered track statuses, where the broken track section has the minimum energy value and the occupied track section provides the maximum value, mainly due to existing noise. With regard to the frequency feature *f_p_*, which represents the largest amplitude *P* in the signal spectrum, it is similar for the free and occupied sections, but it can be clearly distinguished in a broken track section. All three features combined together make it possible to classify the three considered track statuses.

## 4. Experimental Results

**Figure 14:**
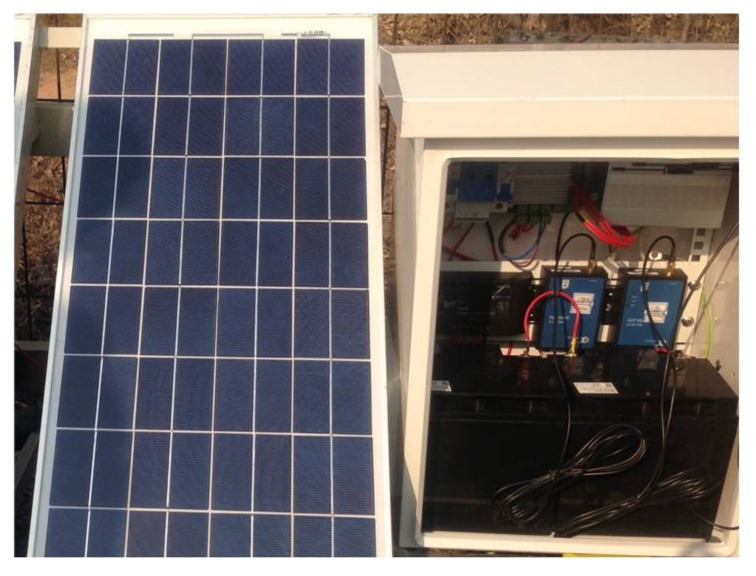
Experimental equipment for each node.

**Figure 15:**
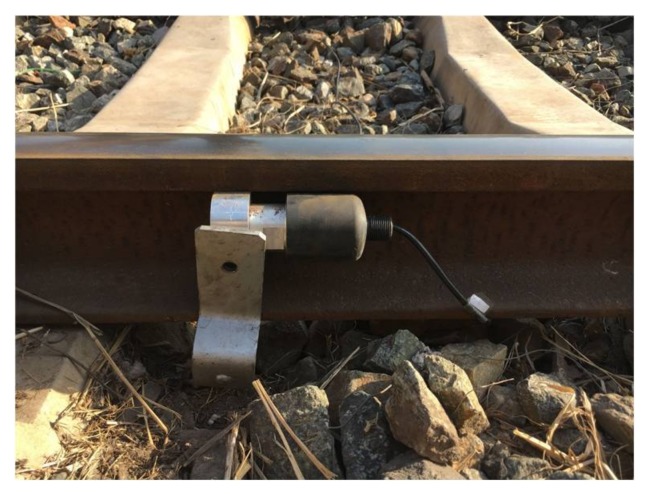
Installation of ultrasonic transducer at rails.

As has been already mentioned, the proposal for the track section status classification was experimentally validated by installing the corresponding equipment on the railway line from Baoji to Chengdu (China). The experimental equipment for every node along the railway line can be observed in Figure 14, whereas the installation of an ultrasonic transducer in the rail is shown in Figure 15. The distance between transmitters and receivers is 1 km, and the weather conditions were relatively dry during testing. The UGW transducers installed along the track were a type of sandwich piezoelectric ceramic UGW transducer, which are customized according to the requirements of transmission distance and the environment of the railway line.

**Figure 16:**
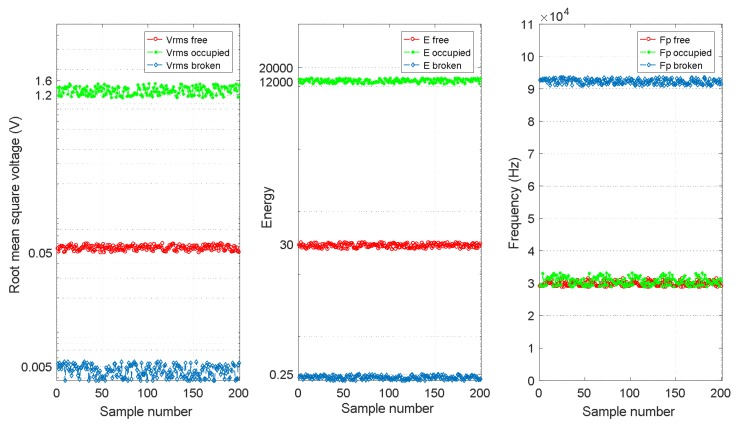
Comparison of three features [*V_RMSi_ E_i_ f_Pi_*] for each different track status for rail no. 2.

**Figure 17:**
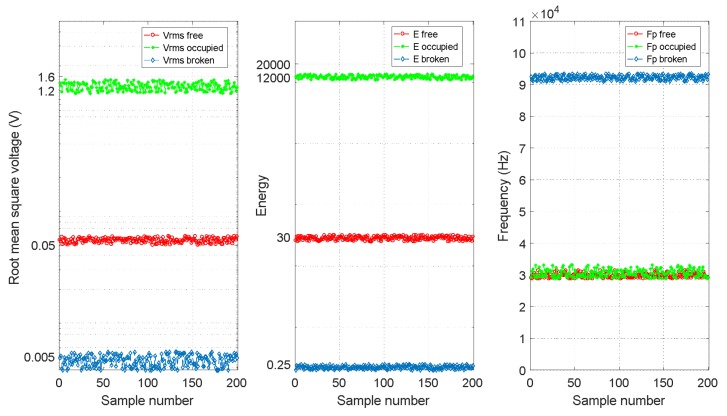
Comparison of three features [*V_RMSi_ E_i_ f_Pi_*] foreach different track status for rail no. 1.

For each section *S_i_*, the three features [*V_RMSi_ E_i_ f_Pi_*] were determined. Therefore, it was possible to analyse the representative collected data for the three track statuses considered and to verify the correctness of the feature extraction proposed in this work. In Figure 16, three different plots allowed us to observe how the three estimated features [*V_RMSi_ E_i_ f_Pi_*] behave for each of the different track statuses considered in rail no. 2. For every feature, 200 representative sample values were available. As can be observed in Figure 16, if the track section is free, all three features [*V_RMSi_ E_i_ f_Pi_*] achieve their ordinary values; for the occupied track status, the features *V_RMSi_* and *E_i_* are much higher than those of the free status, although the frequencies *f_Pi_* are similar to those in the free track section. Finally, if a breakage occurs, the received UGW signals are lost, and *V_RMSi_* and *E_i_* are closed to zero, although the frequency component *f_Pi_* with the largest amplitude *P* under this track status, are higher than those for a free track section. Also, Figure 17 shows that the three features for rail no. 1 behave in a similar way. Thus, by combining the three features together, the three different track statuses can be clearly classified for both rail no. 1 and rail no. 2.

**Figure 18:**
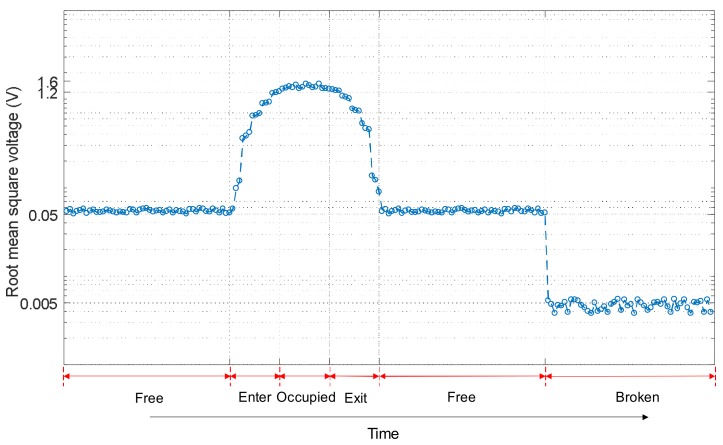
Example of a temporal trend for the feature *V_RMSi_* in a certain track section *S_i_*.

**Figure 19:**
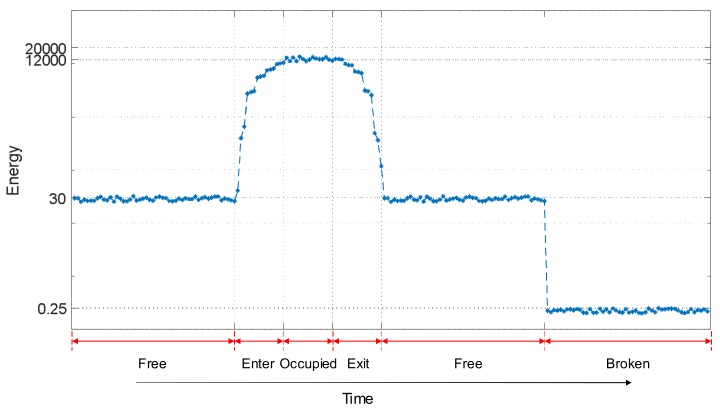
Example of a temporal trend for the feature *E_i_* in a certain track section *S_i_*.

**Figure 20:**
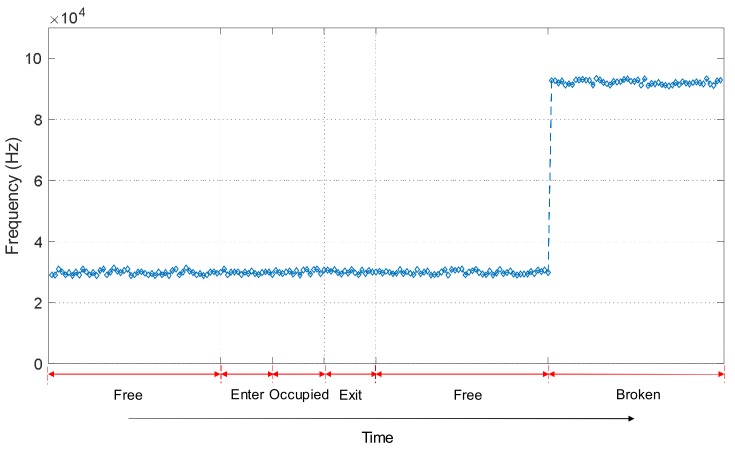
Example of a temporal trend for the feature *f_Pi_* in a certain track section *S_i_*.

In Figure 16 and Figure 17, it is possible to see that the track status can be easily distinguished in a permanent situation. Nevertheless, in some cases, it is also important to determine the status trend: that is, the temporal behaviour for every track section *S_i_* under analysis. Information about the evolution over time of a track section status can be used to distinguish more complex conditions or effects, such as the influence of weather or rail degradation. For that purpose, the temporal trend of the three features [*V_RMSi_ E_i_ f_Pi_*] for the collected experimental samples for a certain track section *S_i_* was analysed and this is shown in Figure 18, Figure 19 and Figure 20. Each figure includes six different statuses over time. As can be observed in Figure 18, if the track section *S_i_* is free, the feature *V_RMSi_* stays at a lower constant value. When there is a train passing along the track section *S_i_*, the values for *V_RMSi_* start to increase as the train approaches, reaching a maximum when the track section is completely occupied by the train. In the same way, *V_RMSi_* decreases gradually as the train exits from the track section *S_i_*, and then it recovers the previous low value for a free status. Finally, when a breakage occurs in section *S_i_*, the feature *V_RMSi_* drops abruptly to almost zero. With regard to the temporal trend of the energy *E_i_* shown in Figure 19, it has a similar evolution as *V_RMSi_*. As far as the frequency component *f_Pi_* is concerned, no matter whether the track section *S_i_* is free, the train is approaching or exiting, or the section *S_i_* is occupied by the train, the maximum frequency component *f_Pi_* maintains an almost constant value around 30 kHz. Nevertheless, this value sharply increases up to *f_Pi_* = 92 kHz when the track status is broken, as can be observed in Figure 20.

**Figure 21:**
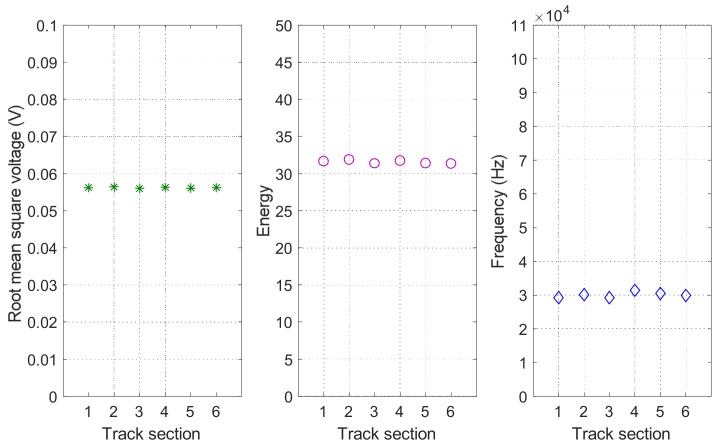
Spatial dependencies of features [*V_RMSi_ E_i_ f_Pi_*] for six neighbouring track sections *S_i_*, *i* = 1, 2, …, 6 in rail no. 2 under free status.

**Figure 22:**
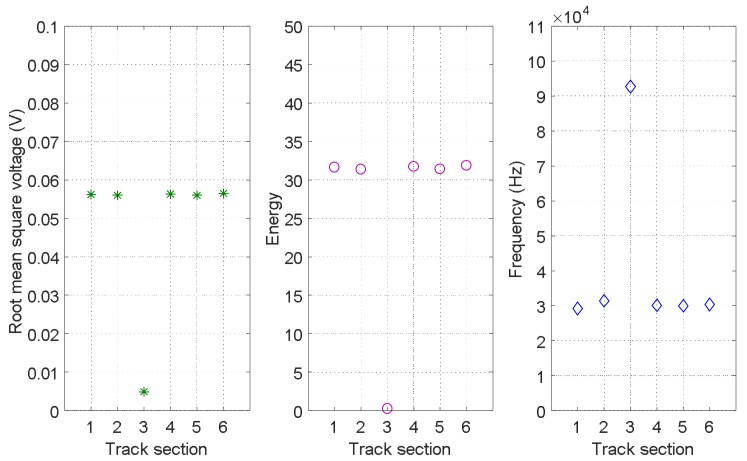
Spatial dependencies of features [*V_RMSi_ E_i_ f_Pi_*] for six neighbouring track sections *S_i_*, *i* = 1, 2, …, 6 in rail no. 2 when section *S*_3_ is broken.

On the other hand, it is important to note that the features from other neighbouring track sections can also provide additional information about the status of the specific track section *S_i_* under analysis, due to the spatial dependencies existing among them. Taking those into account, in the proposed UGW-based monitoring system, the broken status is assumed to influence only one track section, whereas the occupied status is likely to influence all the track sections in the specific moving path of the train. This implies that some information from neighbouring track sections can enhance the status classification for a certain track section *S_i_*. In this work, the three aforementioned features [*V_RMSi_ E_i_ f_Pi_*] for six neighbouring track sections *S_i_*, *i* = 1, 2, …, 6 are considered to explore their spatial dependencies. Firstly, it is assumed all the six track sections in rail 2 are free at the same time, so the three features for the six track sections *S_i_*, *i* = 1, 2, …, 6 present a similar value, as is shown in Figure 21. Secondly, Figure 22 shows an example case, when the status of track section *S*_3_ is broken, whereas the others are free. Compared with the situation in Figure 21, it can be observed that the broken status only affects track section *S*_3_ where it occurs, and not the neighbouring ones.

**Figure 23:**
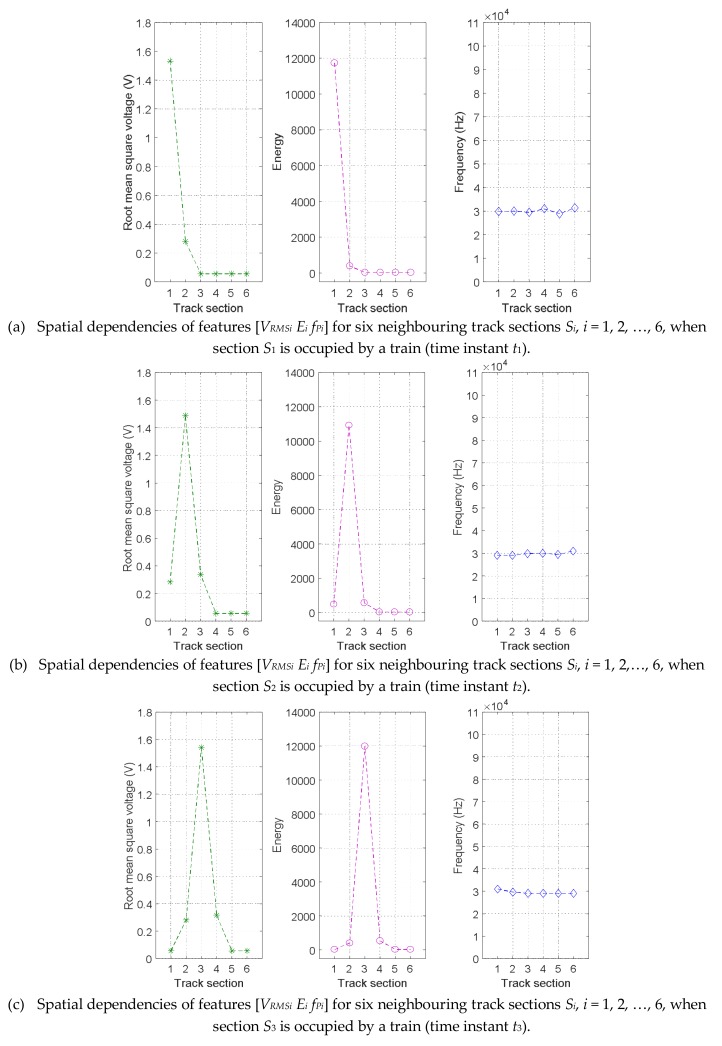

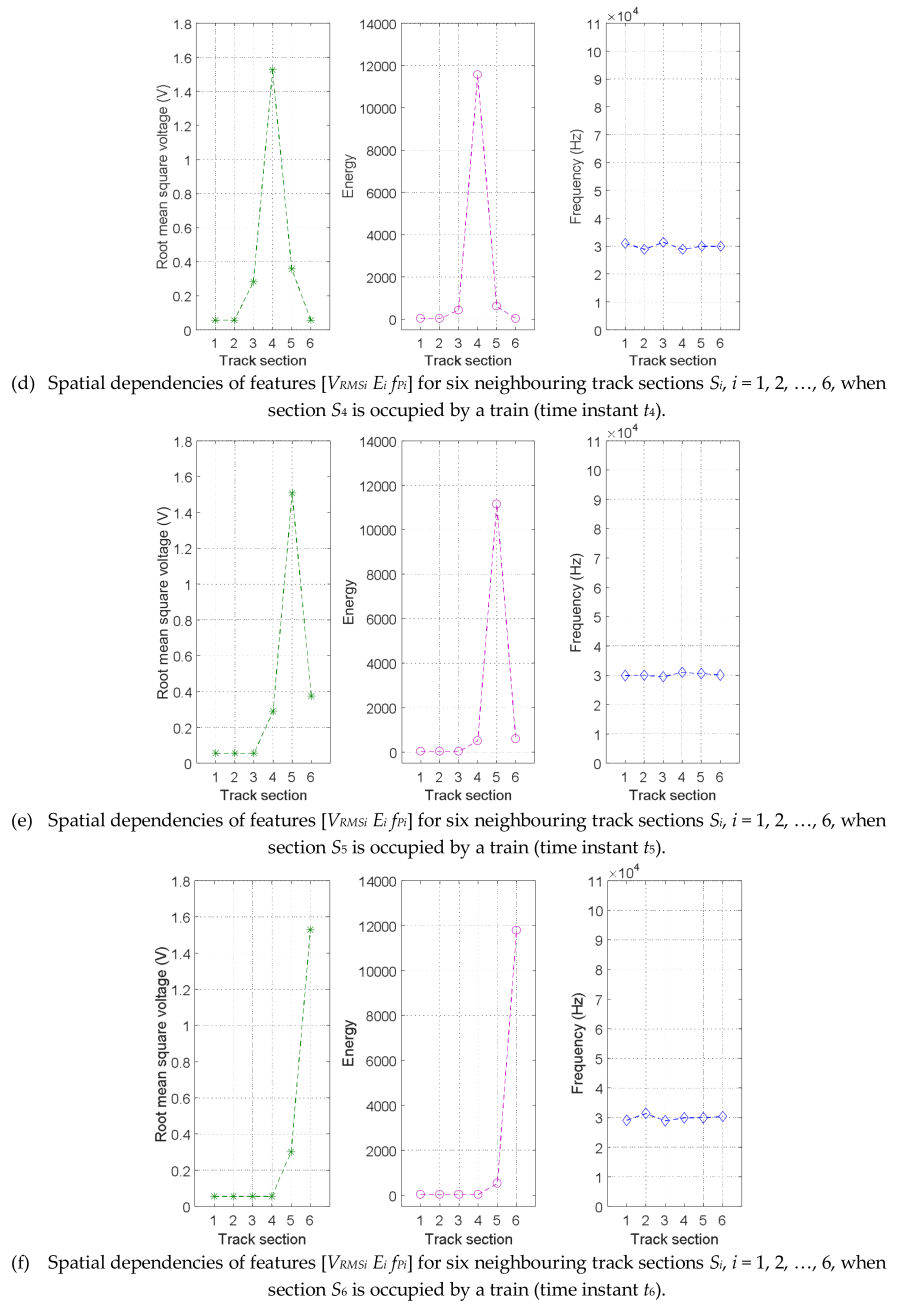
Spatial dependencies of features [*V_RMSi_ E_i_ f_Pi_*] for six neighbouring track sections *S_i_*, *i* = 1, 2, …, 6, in rail no. 2 when a train is moving through them.

Finally, an illustrative situation, at which a train is passing from track section *S*_1_ to track section *S*_6_ in rail 2, is described. The spatial and temporal dependencies of three features [*V_RMSi_ E_i_ f_Pi_*] for this situation can be observed in Figure 23, from (a) to (f), at different times. Firstly, at *t*_1_, the track section *S*_1_ is occupied by a train, so the features *V_RMS_*_1_ and *E*_1_ are the highest. Even though the other five sections are free, due to the influence of the mechanical noise caused by the train, the neighbouring section *S*_2_ has relatively higher values than the others. When the train is moving from track section *S*_2_ to track section *S*_5_ (from *t*_2_ to *t*_5_), no matter which section is occupied, the features *V_RMSi_* and *E_i_* in the corresponding section *S_i_* are the highest, although their immediate neighbours still present relatively higher values than the other three free sections. Finally, when the train arrives in the track section *S*_6_, the feature *V_RMSi_* and *E_i_* for track sections *S_1_* to *S*_4_ are similar, the values for track section *S*_5_ are slightly higher, and the highest values are obtained for track section *S*_6_ (time instant *t*_6_). It is worth noting that no matter where the train is, the frequency components *f_Pi_* for the six track sections *S_i_* always show quite stable behaviour. Figure 23 allows us to conclude that a train occupying a certain track section affects, not only that particular track section, but also the immediate neighbouring sections. This spatial dependency can be used to improve the final track section status classification.

**Figure 24:**
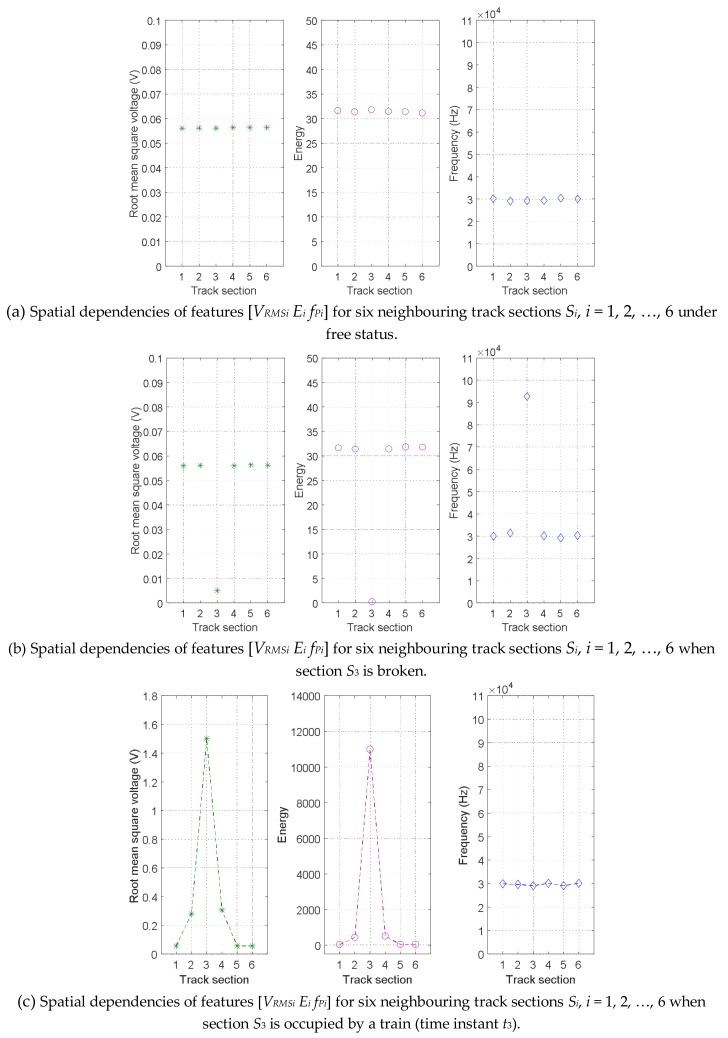
Spatial dependencies of features [*V_RMSi_ E_i_ f_Pi_*] for six neighbouring track sections *S_i_*, *i* = 1, 2, …, 6 in rail no. 1 under three different track statuses.

The same analysis for the spatial dependencies of features [*V_RMSi_ E_i_ f_Pi_*] for six neighbouring track sections in rail no. 1, can also be seen in Figure 24, where similar behaviours as those previously presented for rail no. 2 in Figure 23 were observed. Figure 24. (a) shows the case when all the six track sections *S_i_* are free, whereas in Figure 24. (b) track section *S*_3_ is broken and in Figure 24. (c) track section *S*_3_ is occupied by a train.

## 5. Future Work

Previously, received UGW signals have been analysed for three different track statuses, the associated features have been extracted, and they have been validated by means of experimental tests. These features are thought to be useful as inputs in a future high-level system that carries out the status classification and decision making. In this way, it is intended to apply a recurrent neural network (RNN) [31,32,33], where previous knowledge regarding the dependencies among the three track statuses will not be explicitly integrated. It is, however, important to give the network a structure that enables it to learn these dependencies from data. It is worth noting that for detecting temporal dependencies, a RNN is a natural choice, since the recurrent connections in the network allow memories of past events to be stored.

**Figure 25:**
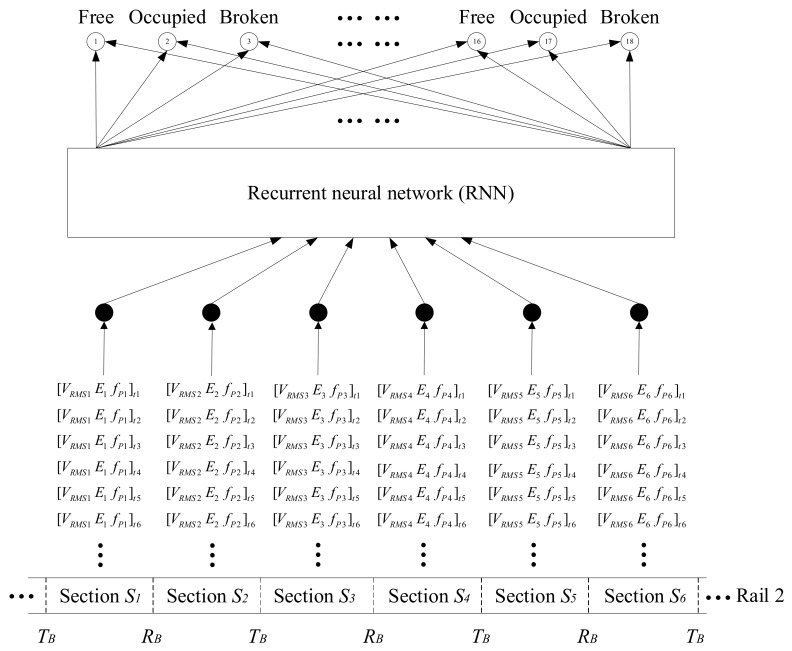
Preliminary approach for the track section status classification process.

Since both rails, no. 1 and no. 2, present the same setup, similar networks can be applied in both cases. This is why the status classification is only detailed for rail no. 2, assuming that it can be extended to rail no. 1. A preliminary approach for the status classification process is depicted in Figure 25, where, six track sections *S_i_*, *i* = {1, 2, …, 6} are involved in the lower part. As was described earlier, the three features [*V_RMSi_ E_i_ f_Pi_*] are calculated for every 20 ms long UGW signal window and for each section *S_i_*, thus becoming the inputs for the RNN. Through the RNN processing, the output layer of the network consists of 18 track status classification units, each three belonging to a section *S*_i_ and denoting the three statuses mentioned before: free, occupied and broken.

It is worth noting that, whether the track section *S*_i_ is free or occupied, the final track section status classification should be unified for both rails, no. 1 and no. 2. Furthermore, in the case that different breakages happen in all the neighbouring sections at the same time, which is actually very improbable, the classification output for rail no. 1 and rail no. 2 may also be different. In this particular case, the final classification result is decided in accordance with safe rail operation.

## 6. Conclusions

A monitoring system based on UGW signals, consisting of the distribution of transmitters and receivers every 1 km along the track, has been described in this work. Every receiver can acquire and process the UGW signals coming from neighbouring transmitters. In order to classify three different track statuses (free, occupied and broken), the main contribution of this work is the extraction of three features from the UGW signals: the RMS value and the energy in the time domain, and the frequency component with the highest amplitude in the frequency domain. The analysis of these three features, together with temporal and spatial dependencies along the successive track sections of a railway line, allow the track section status classification to be carried out correctly. Experimental tests have successfully validated the proposed approach. Finally, a recurrent neural network has been envisaged as a feasible solution to implement a high-level classifier based on the three features already mentioned.
